# Screening of a Library of FDA-Approved Drugs Identifies Several Enterovirus Replication Inhibitors That Target Viral Protein 2C

**DOI:** 10.1128/AAC.02182-15

**Published:** 2016-04-22

**Authors:** Rachel Ulferts, S. Matthijn de Boer, Lonneke van der Linden, Lisa Bauer, Hey Rhyoung Lyoo, Maria J. Maté, Julie Lichière, Bruno Canard, Daphne Lelieveld, Wienand Omta, David Egan, Bruno Coutard, Frank J. M. van Kuppeveld

**Affiliations:** a Department of Infectious Diseases and Immunology, Virology Division, Faculty of Veterinary Medicine, Utrecht University, Utrecht, The Netherlands; b Department of Medical Microbiology, Academic Medical Center, Amsterdam, The Netherlands; c CNRS, AFMB UMR 7257, Marseille, France; d Aix-Marseille Université, CNRS, AFMB UMR 7257, Marseille, France; e Cell Screening Core, Department of Cell Biology, Center for Molecular Medicine, UMC Utrecht, Utrecht, The Netherlands

## Abstract

Enteroviruses (EVs) represent many important pathogens of humans. Unfortunately, no antiviral compounds currently exist to treat infections with these viruses. We screened the Prestwick Chemical Library, a library of approved drugs, for inhibitors of coxsackievirus B3, identified pirlindole as a potent novel inhibitor, and confirmed the inhibitory action of dibucaine, zuclopenthixol, fluoxetine, and formoterol. Upon testing of viruses of several EV species, we found that dibucaine and pirlindole inhibited EV-B and EV-D and that dibucaine also inhibited EV-A, but none of them inhibited EV-C or rhinoviruses (RVs). In contrast, formoterol inhibited all enteroviruses and rhinoviruses tested. All compounds acted through the inhibition of genome replication. Mutations in the coding sequence of the coxsackievirus B3 (CV-B3) 2C protein conferred resistance to dibucaine, pirlindole, and zuclopenthixol but not formoterol, suggesting that 2C is the target for this set of compounds. Importantly, dibucaine bound to CV-B3 protein 2C *in vitro*, whereas binding to a 2C protein carrying the resistance mutations was reduced, providing an explanation for how resistance is acquired.

## INTRODUCTION

The genus Enterovirus (family Picornaviridae) includes many medically and socioeconomically important human pathogens (e.g., poliovirus [PV], coxsackievirus, echovirus, enterovirus A71 [EV-A71], EV-D68, and rhinoviruses [RVs]). The serotypes of the genus Enterovirus are categorized into nine enterovirus species (species A to J) and three rhinovirus species (species A to C). Four of the enterovirus species (species A, B, C, and D) and the three rhinovirus species include serotypes that are known to infect humans. These can cause a wide range of diseases ranging from enteric or respiratory infections, hand-foot-and-mouth disease, or conjunctivitis to acute flaccid paralysis, viral myocarditis, fulminant pancreatitis, or aseptic meningitis. Infections are often self-limiting but can result in severe complications that are fatal in some rare cases. In addition, preexisting medical conditions can be exacerbated. For example, rhinoviruses have been shown to aggravate asthma and chronic obstructive pulmonary disease (COPD) (1–3).

No approved antiviral therapeutics exist to date, and treatment remains limited to supportive care. The highly successful poliovirus vaccines are the only vaccines against human enteroviruses, and with the current total number of serotypes exceeding several hundred, vaccine development against all enteroviruses is unlikely to be a realistic option. In summary, there is a need for the development of new antiviral drugs.

Enteroviruses are small, nonenveloped viruses with icosahedral capsids. The positive-sense, single-stranded RNA genome of ∼7.5 kb encodes a single large polyprotein that is autocatalytically processed into four structural (VP1 to VP4) and seven nonstructural (2A to 2C and 3A to 3D) proteins as well as several processing intermediates. Several of these viral proteins have been identified as potential targets of antivirals (reviewed in reference 4). Particularly, those with (proven or predicted) enzymatic activity, like 3C, one of the two proteases involved in autocatalytic processing; 3D, the RNA-dependent RNA polymerase; and 2C, a putative helicase, are under investigation as targets of antivirals. Furthermore, compounds that bind to the virus capsid and thereby interfere with virus entry and/or uncoating have been developed (reviewed in reference 4). Alternatively, host factors, i.e., cellular proteins usurped by these obligate intracellular pathogens, have been shown to be feasible targets for antiviral intervention (5–8).

Classical drug development is a costly and laborious process. Drug repurposing, that is, the discovery of new indications for existing drugs, can simplify this process. Here drugs with well-established safety profiles are screened for their inhibitory effect on enterovirus infection.

By screening the Prestwick Chemical Library of approved drugs for novel enterovirus inhibitors, we detected the antiviral activity of five drugs, fluoxetine, pirlindole, dibucaine, zuclopenthixol, and formoterol. Fluoxetine, dibucaine, and zuclopenthixol were also identified as inhibitors of coxsackievirus B3 (CV-B3) in a Prestwick Chemical Library screen by Zuo et al. (9), but except for fluoxetine, which we identified in an independent screen and showed to inhibit EV-B and EV-D species by targeting viral protein 2C (10), the antiviral activity of these drugs has not been further investigated. Hence, their spectrum of antiviral activity against other enteroviruses as well as their target are unknown. Also, details of the antiviral activities of formoterol, which was recently identified as an inhibitor of human rhinovirus 14 (11), and pirlindole mesylate, which, to our knowledge, has not previously been identified as an inhibitor of an enterovirus, are unknown. Therefore, we further characterized the antiviral activities of dibucaine, zuclopenthixol, pirlindole mesylate, and formoterol. We show that all four compounds acted at the genome replication stage. Although of diverse chemical structures, three of these compounds, pirlindole, dibucaine, and zuclopenthixol, appeared to act on viral protein 2C, as mutations in this protein conferred resistance to all three compounds. Furthermore, direct binding of dibucaine to protein 2C was observed. These three compounds showed the strongest antiviral effect against EV-B and EV-D members. In contrast, formoterol, which does not target 2C, inhibited the replication of all enteroviruses and rhinoviruses tested. It seems unlikely that the known cellular target of formoterol, i.e., the β2 receptor, accounts for the antiviral mechanism, as other compounds that act on this target did not show antiviral activity. Unfortunately, no formoterol-resistant viruses could be recovered; hence, the mechanism of action of this compound remains to be established.

## MATERIALS AND METHODS

### Cells and reagents.

Buffalo green monkey (BGM) kidney cells and HeLa R19 cells were grown at 37°C with 5% CO_2_ in Dulbecco's modified Eagle medium (DMEM; Gibco) supplemented with 5% fetal bovine serum and antibiotics. Pirlindole mesylate was purchased from Santa Cruz Biotechnology; tetrindole mesylate and moclobemide were purchased from Tocris; and dibucaine hydrochloride (HCl), oxethazaine, guanidine hydrochloride (GuHCl), and dipyridamole were purchased from Sigma-Aldrich. Pirlindole mesylate, dibucaine HCl, and GuHCl were dissolved in water. The other compounds were dissolved in dimethyl sulfoxide (DMSO).

### Viruses and virus infections.

CV-B3 (strain Nancy) was obtained by transfection of BGM cells with runoff RNA transcripts of the full-length infectious clone p53CB3/T7 linearized with MluI (12). Green fluorescent protein (GFP)- or Renilla luciferase (Rluc)-expressing CV-B3 was likewise obtained (13). Encephalomyocarditis virus (EMCV, strain mengovirus) was obtained in a similar manner by transfection of RNA derived from cDNA clone pM16.1 into BHK-21 cells (14). EV-D68, EV-A71 (BrCr), CV-A16 (G-10), and CV-A21 (Coe) were obtained from the National Institute for Public Health and Environment (RIVM, The Netherlands). The poliovirus 1 Sabin reference strain was obtained from J. Martin (NIBSC, United Kingdom). Rhinoviruses A2 and B14 were supplied by Joachim Seipelt (Medical University of Vienna, Vienna, Austria). Cells were incubated with virus at the indicated multiplicity of infection (MOI) for 30 min at 37°C. The inoculum was then replaced with medium with or without the compound, and cells were incubated for the indicated times at 37°C. For analysis of virus titers, the cells were freeze-thawed three times to release intracellular virus particles, and virus titers were determined by endpoint titration according to the method of Reed and Muench and expressed as 50% cell culture infective doses (CCID_50_) per milliliter (15). Renilla luciferase activity was determined by using the Renilla luciferase assay kit (Promega) according to the manufacturer's instructions.

Cell viability was assessed by using 3-(4,5-dimethylthiazol-2-yl)-2,5-diphenyltetrazolium bromide (MTT; Sigma) as described previously (16). Briefly, 0.2 mg/ml MTT in DMEM with 2% fetal calf serum (FCS) was added to the cells, and the cells were incubated for 1 h at 37°C. The supernatant was then removed, the precipitate was dissolved in DMSO, and the optical density at 570 nm (OD_570_) was determined. The measured values were used to calculate cell viability ranging from 0% (empty wells) to 100% (untreated cultures). The 50% cytotoxic concentration (CC_50_) was defined as the concentration of compound that resulted in a 50% reduction of cell viability and was calculated by using logarithmic interpolation.

### Replicon assay and mutant viruses.

Subconfluent BGM cells were transfected with 15 ng *in vitro*-transcribed RNA from MluI-linearized CV-B3 wild-type (wt) pRib-LUC-CB3/T7 (17) or p53CB3/T7 and CV-B3 mutants 2C(A224V,I227V,A229V) (18) or 3A(H57Y) (19) per well of a 96-well plate by using 0.075 μl Lipofectamine 2000 (Invitrogen) and incubated for 30 min at 37°C. The transfection mix was then replaced with medium with or without the compound. For the measurement of firefly luciferase (Fluc) activity, cell layers were washed with phosphate-buffered saline (PBS) and lysed in lysis buffer (50 mM tricine-HCl [pH 8.0], 100 μM EDTA, 2.5 mM MgSO_4_, 10 mM dithiothreitol [DTT], 1% Triton X-100), and activity was measured by using firefly luciferase assay buffer (50 mM tricine-HCl [pH 8.0], 100 μM EDTA, 2.5 mM MgSO_4_, 10 mM DTT, 1.25 mM ATP, 12.5 μM d-luciferin). Virus titers were determined as described above.

### Small-molecule screen.

Each compound from the Prestwick Chemical Library (5 mM) was added at a final concentration of 10 μM per well 1 day after seeding of HeLa R19 cells (2,800 cells per well) in 384-well plates by using a Caliper Sciclone liquid-handling robot. At 1 h posttreatment, cells were infected with CV-B3–GFP to achieve an average infection of 20%. At 6 h postinfection (p.i.), cells were fixed with 4% paraformaldehyde, and nuclei were counterstained with DAPI (4′,6-diamidino-2-phenylindole). Six sites in each well were imaged at a ×10 magnification for GFP fluorescence- and DAPI-stained cells with a Thermo ArrayScan VTi automated microscope. The percentage of GFP-positive cells in the DAPI-positive population was calculated by using automated image analysis (Thermo Cellomics Target Activation Bioapplication). The raw data were loaded into a Web-based high-content data-mining package, HC StratoMineR (https://hcstratominer.umcutrecht.nl/) (W. Omta and D. Egan, unpublished data). Significant hits were identified based on the percentage of infection and the fold reduction of infection over the control for each well. Wells that presented a >10% reduction in the number of DAPI-positive cells compared to DMSO-treated HeLa R19 cells were considered to have suffered from cytotoxicity and were eliminated from further analysis.

### Protein 2C expression and isothermal titration calorimetry.

The DNA fragment coding for 2C Del36 of CV-B3 (amino acids 37 to 329) was cloned downstream of a cleavable thioredoxin-hexahistidine tag. Mutations were introduced into the 2C coding sequence by using PCR-based site-directed mutagenesis.

The proteins were produced in Escherichia coli T7 Express (New England BioLabs) at 17°C. Purification of the protein and tag removal were performed under nondenaturing conditions as previously described (10, 20). The final size-exclusion chromatography step was performed with a solution containing 10 mM HEPES and 300 mM NaCl (pH 7.5). The binding of dibucaine to wild-type (wt) and A224V/I227V/A229V mutant 2C proteins was measured by isothermal titration calorimetry (ITC) using a MicroCal iTC200 instrument (Malvern). Experiments were carried out at 20°C in a solution containing 10 mM HEPES, 300 mM NaCl, and 1% DMSO (pH 7.5). The protein concentrations in the cell were 90 μM and 150 μM for the wt and mutant proteins, respectively, whereas the dibucaine concentration in the syringe was 1 mM. Heats of dilution were measured by injecting the ligand into the protein solution. Titration curves were fitted by using MicroCal Origin software, assuming one set of sites, and enthalpy changes (Δ*H*), dissociation equilibrium constants (*K_d_*), and stoichiometry were extracted.

## RESULTS

### Compound screen.

**FIG 1:**
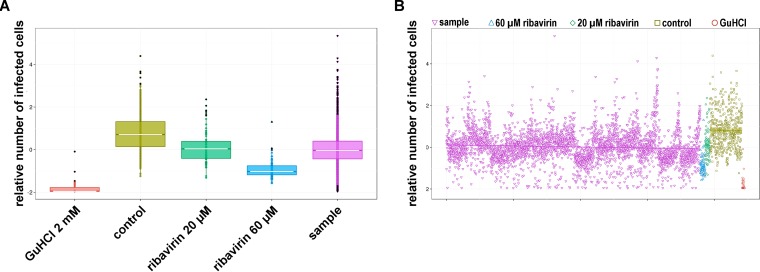
Screening controls and overall screening data. (A) Data showing levels of infection based on intensity of GFP staining. Shown are box-and-whisker plots for screening controls and samples. All data were normalized against the negative controls in each plate. The lot displays the median, interquartile ranges (IQR), and outliers (outside 1.5 times the interquartile range). (B) Scatter plot showing data for all individual samples and controls. The regression line was plotted based on a least-squares criterion.

To identify compounds with antiviral activity against enteroviruses, we screened the Prestwick Chemical Library, a library consisting of 1,200 approved drugs (U.S. Food and Drug Administration, European Medicines Agency, or other agency), for compounds that inhibit the replication of CV-B3 by using the CV-B3–GFP reporter virus. This reporter virus encodes GFP upstream of the capsid coding region. The intracellular GFP expression levels in CV-B3–GFP-infected cells thus correlate with virus replication (13). Cells were pretreated with 10 μM compound and infected with virus, and GFP expression was analyzed at 6 h p.i. This setup allows the detection of compounds that affect entry and replication but not of those that affect assembly. The known enterovirus inhibitors ribavirin (20 μM and 60 μM) and guanidine hydrochloride (GuHCl) (2 mM) were included as positive controls. The screen was performed twice with three replicates of each library compound. Ribavirin and GuHCl strongly decreased the mean average enhanced GFP (EGFP) fluorescence intensity, consistent with their known antiviral activity (Fig. 1A and B). Compounds that reduced the median average fluorescence intensity >20-fold and resulted in <10% reduction of the median number of DAPI-positive cells (as a measure of cell viability) in both screens were considered primary hits. These compounds were pirlindole mesylate, fluoxetine hydrochloride, formoterol fumarate, dibucaine, and zuclopenthixol hydrochloride (Table 1). The identified drugs are of diverse structures, belonging to diverse chemical classes, and are known to target different cellular factors. Formoterol is an agonist of β2 adrenergic receptors (β2-ARs) that is currently used as a bronchodilator for the treatment of asthma. Dibucaine is in use as a topical anesthetic that acts through the inhibition of voltage-activated sodium channels. Pirlindole is an inhibitor of monoamine oxidase A (MAO-A) (21), an enzyme involved in the metabolism of monoamines, including serotonin, melatonin, adrenaline, and noradrenaline. Zuclopenthixol is an antagonist of D2 dopamine receptors and used for the treatment of psychotic disorders. Fluoxetine HCl (Prozac) is a selective serotonin reuptake inhibitor used to treat depression. We and others previously identified fluoxetine in independent screens as an inhibitor of EV-B and EV-D members, and we showed that the compound acts on viral protein 2C (9, 10). Thus, these four compounds, formoterol, pirlindole mesylate, dibucaine, and zuclopenthixol, were analyzed further.

**TABLE 1:**
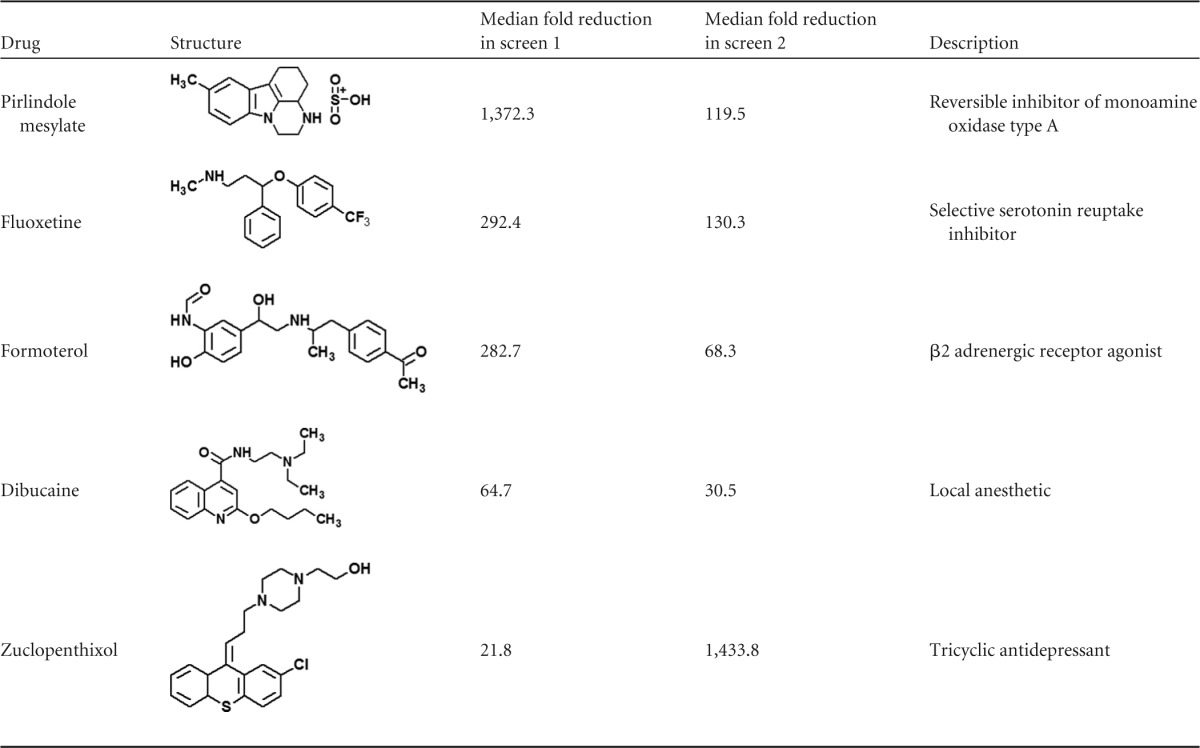
Structural formulae of hits

### Dibucaine, pirlindole, and zuclopenthixol inhibit CV-B3 replication.

To confirm the antiviral activity of the remaining hits and to exclude batch-specific effects, we reordered formoterol fumarate, dibucaine HCl, and pirlindole mesylate from a different supplier (see Materials and Methods). Zuclopenthixol was unfortunately not commercially available. We therefore retested the stock from the compound library for antiviral activity.

For retesting, we used a Renilla luciferase-expressing CV-B3 reporter virus (CV-B3–Rluc). This virus encodes Renilla luciferase upstream of the capsid coding region, and the intracellular luciferase activity in infected cells can be used as a sensitive measure of virus replication (13). To exclude that a reduction of luciferase activity is due to direct inhibition of Renilla luciferase activity or a general inhibition of cellular translation, a separate set of cells was transfected with *in vitro*-transcribed, capped RNA encoding Renilla luciferase (Rluc mRNA) and treated with the same concentrations of compounds. Adverse effects of the compounds on cellular viability were analyzed by using the well-established 3-(4,5-dimethylthiazol-2-yl)-2,5-diphenyltetrazolium bromide (MTT) reduction assay.

**FIG 2:**
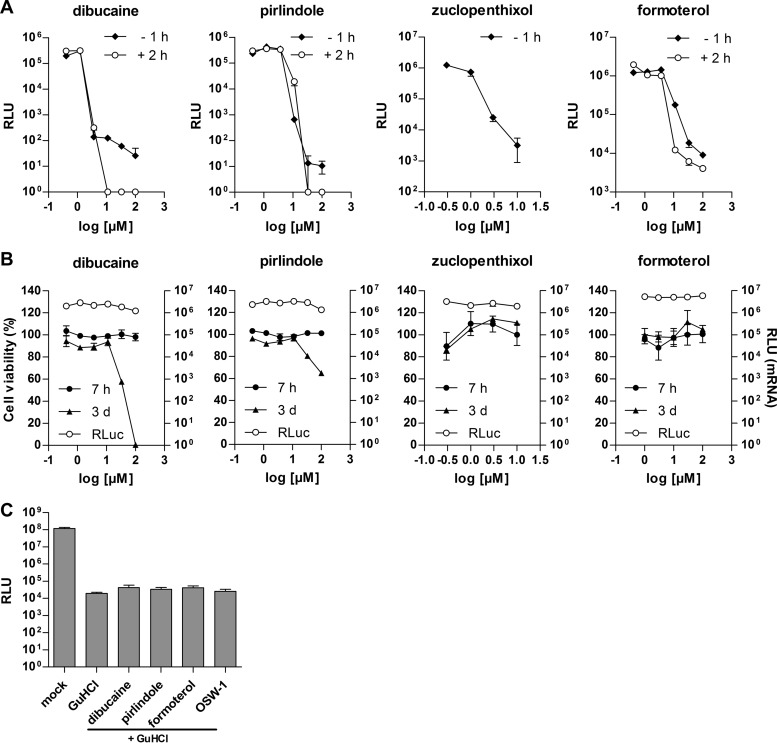
Antiviral activities of dibucaine, pirlindole, zuclopenthixol, and formoterol. (A) HeLa R19 (dibucaine, pirlindole, and formoterol) or BGM (zuclopenthixol) cells transfected with *in vitro*-transcribed CV-B3 replicon RNA were treated with various concentrations of the indicated compounds either 1 h prior to transfection (−1 h) or 2 h after transfection (2 h). (B) Cells were transfected with *in vitro*-transcribed RNA encoding Rluc and treated with compound as described above for panel A. Renilla luciferase values were determined 6 h after transfection. For cell viability measurement by MTT assays, cells (uninfected and untransfected) were treated with various concentrations of the indicated compounds, and cell viability was assessed at 7 h and 3 days posttreatment by using an MTT assay as described in Materials and Methods. (C) BGM cells were infected with CV-B3–Rluc for 30 min, after which the inoculum was replaced with medium containing GuHCl in combination with the indicated drugs. The inhibitor concentrations were 2 mM GuHCl, 10 μM pirlindole, 5 μM dibucaine, 10 μM formoterol, and 10 nM OSW-1. Luciferase values were determined at 6 h p.i. RLU, relative luciferase units.

Cells were preincubated with various concentrations of the compounds for 1 h and infected with CV-B3–Rluc or transfected with Rluc mRNA. Renilla luciferase activity was analyzed at 6 h p.i. A dose-dependent decrease of luciferase activity in CV-B3–Rluc-infected cells was observed in the presence of dibucaine and zuclopenthixol at concentrations of >1 μM and in the presence of formoterol and pirlindole at concentrations of >3 μM (Fig. 2A). The observed reduction of luciferase activity was not due to a direct inhibition of Renilla luciferase activity or a direct effect on translation, as no effect on Renilla luciferase activity was observed in cells transfected with Rluc mRNA at these compound concentrations (Fig. 2B). No adverse effects on cellular viability were observed in the MTT assay up to the highest concentration tested (100 μM for formoterol, dibucaine, and pirlindole and 30 μM for zuclopenthixol) within the time span used for the antiviral assay (7 h) (Fig. 2B). Only after 3 days of incubation reduced cell viability was observed at high concentrations for dibucaine and pirlindole, while no toxicity was observed for formoterol and zuclopenthixol.

To provide further evidence that the compounds do not affect viral, i.e., internal ribosome entry site (IRES)-dependent, translation, we infected cells with CV-B3–Rluc in the presence of the drugs together with GuHCl, a well-known inhibitor of enterovirus RNA replication, allowing us to specifically assess potential effects of the incoming viral RNA on translation efficiency. As a control, we included OSW-1, which inhibits enterovirus replication by targeting the host factor oxysterol-binding protein but has no effect on translation (8). Measurement of the Renilla luciferase activity showed that neither dibucaine, pirlindole, nor formoterol caused any reduction in luciferase levels (Fig. 2C), confirming that these drugs do not affect viral translation efficiency. Thus, we concluded that formoterol, dibucaine, pirlindole, and zuclopenthixol exhibit antiviral activity against CV-B3.

### Antiviral spectra of dibucaine, pirlindole, and formoterol.

**FIG 3:**
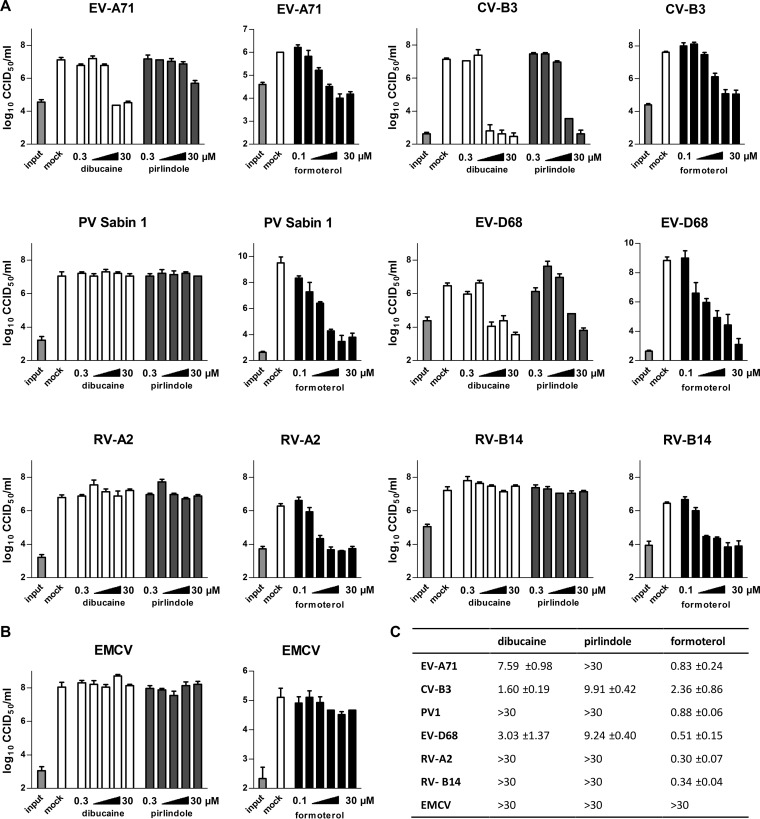
Effects of dibucaine, pirlindole, and formoterol on selected picornaviruses in a single-cycle assay. The antipicornaviral spectra of dibucaine, pirlindole, and formoterol were analyzed in a single-cycle assay using a panel of representative picornaviruses. HeLa R19 (EV-D68, RV-A2, and RV-B14) or BGM (all other viruses) cells were infected with the indicated virus at an MOI of 10 CCID_50_ per cell and treated with various concentrations of dibucaine or pirlindole (0.3, 1, 3, 10, or 30 μM) or formoterol (0.1, 0.3, 1, 3, 10, or 30 μM) or mock treated. Viruses were harvested at 8 h p.i., and virus titers were determined by endpoint titration. Shown are the means and standard deviations of data from three replicates. (A) Enteroviruses; (B) cardiovirus; (C) calculated EC_50_ values (micromolar).

To analyze whether the compounds act on other related viruses, we tested their activity against representative serotypes of different enterovirus species in a single-cycle assay. The panel of viruses consisted of EV-A71 as a representative of enterovirus species A (EV-A), CV-B3 for EV-B, poliovirus Sabin 1 (EV-C), EV-D68 (EV-D), rhinovirus (RV) A2 (RV-A), and RV-B14 (RV-B). We also included encephalomyocarditis virus (EMCV) from the genus Cardiovirus. Cells were infected with the indicated viruses and treated with different concentrations of dibucaine, pirlindole, and formoterol. Due to the scarcity of zuclopenthixol, this compound could not be included in this analysis. Virus titers were determined at 8 h p.i. Pirlindole efficiently inhibited EV-D68 and CV-B3 and showed moderate activity against EV-A71 but not against any of the other viruses (Fig. 3A and B). Dibucaine was similarly active against CV-B3 and EV-D68 and was also active against EV-A71 albeit with lower potency than against CV-B3 and EV-D68 (Fig. 3A). Replication of PV, RV-A2, RV-B14 (Fig. 3A), and EMCV (Fig. 3B) was not affected by either compound at the concentrations tested. In addition to CV-B3, formoterol inhibited the replication of all other enteroviruses tested, i.e., EV-A71, RV-A2, and RV-B14 (Fig. 3A), but not the cardiovirus EMCV (Fig. 3B). This indicates that formoterol is a panenterovirus inhibitor.

### Dibucaine, pirlindole, and formoterol act on the genome replication stage.

We then tested whether the compounds act on or after the entry of the virus into the cell. Cells were infected with CV-B3–Rluc, and the indicated concentrations of the compound were added at 2 h p.i. Inhibition of Renilla luciferase activity was observed (Fig. 2A) with 50% effective concentrations (EC_50_s) similar to those calculated for compound addition prior to infection (EC_50_ of 1.7 μM for formoterol, EC_50_ of 1.3 μM for dibucaine, and EC_50_ of 7.7 μM for pirlindole). Within the first 2 h of infection, the virus attaches to the cell and is taken up, and the RNA genome is released and translated, while replication of the genome remains undetected (22). The finding that the compounds are still able to reduce Renilla luciferase activity when added at 2 h p.i. suggests that the compounds inhibit the genome replication phase.

**FIG 4:**
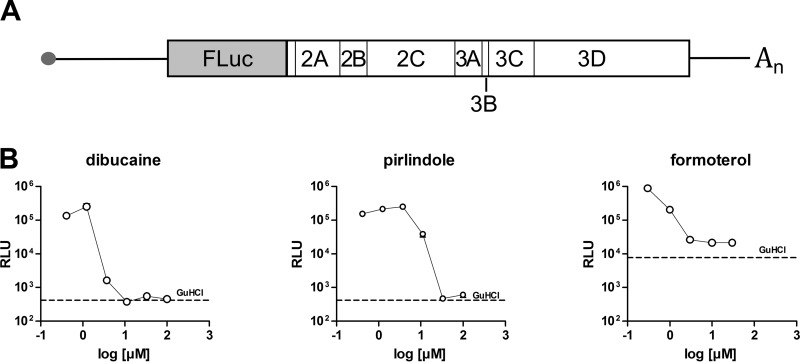
Dibucaine, pirlindole, and formoterol act on the RNA replication stage. (A) Schematic representation of the genome organization of the CV-B3–Fluc subgenomic replicon. (B) BGM cells were transfected with *in vitro*-transcribed CV-B3–Fluc replicon RNA and treated with the indicated concentrations of dibucaine, pirlindole, or formoterol or 2 mM GuHCl 2 h later. Firefly luciferase activity was measured 6 h after transfection. The line indicates the level of luciferase activity measured in the presence of 2 mM GuHCl.

To confirm that pirlindole, dibucaine, and formoterol act at the replication stage, we used the CV-B3–Fluc subgenomic replicon assay (23). In this replicon, the structural proteins, encoded in the P1 region, are replaced with the gene encoding the firefly luciferase (Fig. 4A). For each translated genome, a luciferase protein is also produced, and the measured luciferase activity therefore correlates with viral RNA replication. The *in vitro*-transcribed RNA is delivered into the cells by lipofection, bypassing natural capsid-mediated entry. The addition of either dibucaine, pirlindole, or formoterol resulted in dose-dependent decreases of luciferase activity, with EC_50_s similar to those observed previously (2.6, 1.5, and 8.5 μM for formoterol, dibucaine, and pirlindole, respectively) (Fig. 4B), confirming that the compounds act on genome replication.

### Known targets of the compounds do not explain the observed antiviral activity.

**FIG 5:**
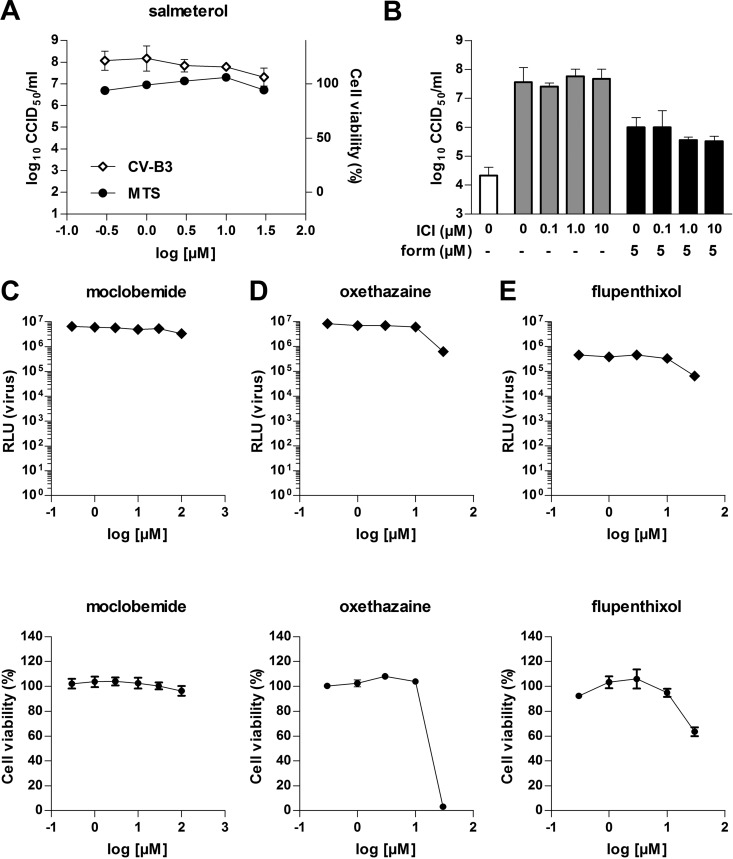
Other compounds that inhibit known cellular targets have no effect on virus replication. (A) Cells were infected with CV-B3 or mock infected and treated with the indicated concentrations of salmeterol. At 8 h p.i., virus titers were determined by endpoint titration. Cell viability was measured by using the MTS assay. (B) Cells were infected with CV-B3 and treated with the indicated concentrations of the β2-AR antagonist ICI-118,551 (ICI) in the absence (DMSO) or presence of 5 μM formoterol (form). At 8 h p.i., virus titers were determined by a limiting-dilution assay. (C) Cells were infected with CV-B3–Rluc or mock infected and treated with various concentrations of the indicated compounds. At 6 h p.i., Renilla luciferase activity was measured. Cell viability was determined by using the MTT assay.

To test if the described targets of the compounds are responsible for the observed antiviral activity, we tested whether alternative compounds that act on these targets also possess antiviral activity. Cells were infected with CV-B3(-Rluc) and treated with various concentrations of the indicated compounds. Salmeterol, like formoterol, a long-acting agonist of β2 adrenergic receptors, did not inhibit virus replication up to a concentration of 33 μM, considerably higher than the EC_50_ of formoterol (Fig. 5A). Also, ICI-118,551, a potent antagonist of β2 adrenergic receptors, did not counteract the antiviral activity of formoterol, further supporting the idea that activation of β2 adrenergic receptors is not the mechanism of enterovirus inhibition (Fig. 5B).

Moclobemide, like pirlindole, a potent inhibitor of MAO-A, did not affect virus replication (EC_50_ of >100 μM), excluding inhibition of MAO-A as a mechanism of virus inhibition of this compound (Fig. 5C).

Oxethazaine, like dibucaine, an inhibitor of voltage-gated sodium channels, did not inhibit virus replication at concentrations of up to 10 μM (Fig. 5D). Higher concentrations could not be tested due to toxicity. Thus, based on this assay, we could neither discount nor prove that inhibition of voltage-gated sodium channels causes the antiviral activity of dibucaine.

Flupenthixol, like zuclopenthixol, an antagonist of D2 dopamine receptors, possessed no antiviral activity at concentrations of up to 10 μM and was toxic at higher concentrations (Fig. 5E).

### Mutations in viral protein 2C confer resistance to dibucaine, pirlindole, and zuclopenthixol.

**FIG 6:**
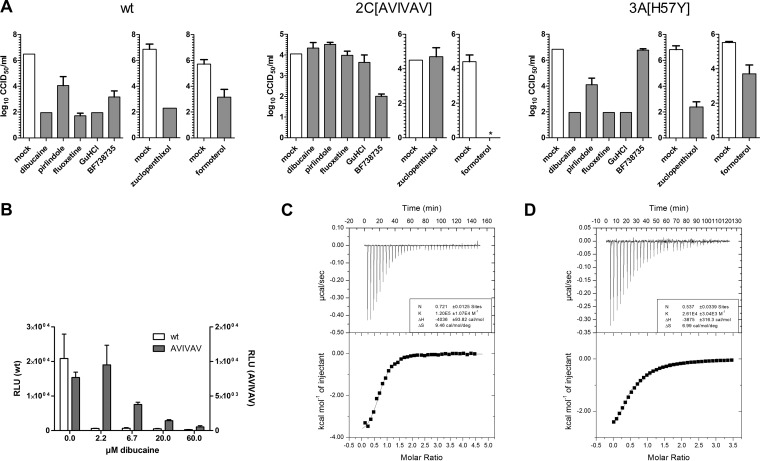
Mutations in viral protein 2C confer resistance to dibucaine, pirlindole, and zuclopenthixol. (A) BGM cells were transfected with *in vitro*-transcribed full-length genomic RNA of wt CV-B3 or the 2C(A224V,I227V,A229V) (AVIVAV) or 3A(H57Y) mutant virus and treated with 5 μM dibucaine, 10 μM pirlindole, 10 μM fluoxetine, 2 mM GuHCl, 1 μM the PI4KIIIβ inhibitor BF738735, 3 μM zuclopenthixol, or 10 μM formoterol or mock treated. Cells were lysed at 8 h p.i., and the virus titer was determined by endpoint titration. Titers are displayed as log CCID_50_ per milliliter, and means and standard deviations were calculated from three replicates. *, below the detection level. (B) Cells were transfected with *in vitro*-transcribed wt or 2C(A224V,I227V,A229V) replicon RNA and treated with the indicated amounts of dibucaine or mock treated. Firefly luciferase activity was determined at 7 h posttransfection. (C and D) Binding of dibucaine to wt (C) and mutant (D) 2C Del36 proteins was analyzed by ITC. Representative examples of the raw data are depicted at the top, and the integrated data are depicted at the bottom. Data are shown fitted to a one-site binding model.

As a first step to identify the antiviral target of the compounds, we studied their effect on the replication of CV-B3 mutant viruses that we previously selected for resistance against other inhibitors of genome replication. CV-B3 carrying a mutation in 3A [3A(H57Y)], which confers resistance to phosphatidylinositol 4-kinase III beta (PI4KIIIβ) inhibitors (e.g., PIK93, enviroxime, GW5074, and BF738735) (24) and inhibitors of oxysterol-binding protein (itraconazole and OSW-1) (8), or in 2C [2C(A224V,I227V,A229V)], which confers resistance to fluoxetine, TBZE-029, and GuHCl (10, 18), was tested for cross-resistance to formoterol, dibucaine, pirlindole, and zuclopenthixol. Cells were transfected with *in vitro*-transcribed RNA of (i) wt CV-B3, (ii) the 3A(H57Y) mutant virus, or (iii) the 2C(A224V,I227V,A229V) mutant virus and treated with 10 μM pirlindole, 5 μM dibucaine, 3 μM zuclopenthixol, or 10 μM formoterol or mock treated. The PI4KIIIβ inhibitor BF738735 (named compound 1 in reference 5) or the 2C-targeting compounds GuHCl and fluoxetine were included as controls. Virus titers were determined at 8 h posttransfection. The 2C mutant virus in the presence of pirlindole, dibucaine, or zuclopenthixol replicated to titers similar to those in untreated cells (Fig. 6A). Also, GuHCl- and fluoxetine-treated 2C mutant virus replicated to similar titers. Conversely, titers of the 3A(H57Y) mutant virus were reduced in the presence of pirlindole, dibucaine, zuclopenthixol, and formoterol, indicating that this mutation does not confer resistance to either of these compounds, while this virus, as previously described (5), was not sensitive to the PI4KIIIβ inhibitor BF738735 (Fig. 6A). In summary, neither of the two tested mutants conferred resistance to formoterol, while the mutations in 2C conferred resistance to pirlindole, dibucaine, and zuclopenthixol, which is suggestive of a mode of action for these three compounds that is similar to that for GuHCl and fluoxetine.

We then tested the sensitivity of the 2C mutant to dibucaine over a range of concentrations. Cells were transfected with *in vitro*-transcribed wt or 2C mutant replicon RNA and treated with various concentrations of dibucaine or mock treated. Firefly luciferase values were determined at 7 h posttransfection. Luciferase values in mock-treated cells transfected with the 2C mutant replicon were half of those in wt replicon-transfected cells (Fig. 6B). This is consistent with the described growth phenotype of this mutant (18) and was also observed in the cross-resistance experiment (Fig. 6A, compare mock-treated wt and mutant full-length RNA-transfected cells). Nevertheless, as described above, replication of the mutant was less affected than that of the wt, although resistance was only partial, and full inhibition was observed at concentrations of >20 μM.

As neither of the two tested virus mutants was resistant to formoterol, we tried to obtain resistant viruses by growing the virus in the presence of various concentrations of the compound. In spite of several attempts, no viruses with reduced sensitivity were obtained.

### Dibucaine binds to protein 2C *in vitro*.

We analyzed the binding of dibucaine and pirlindole to protein 2C *in vitro* using isothermal titration calorimetry (ITC). The production and purification of full-length 2C proteins usually lead to polydisperse preparations, which are not amenable to ITC experiments (25). By removing the 36 N-terminal amino acids containing the amphipathic helix, we generated a monomeric protein (CV-B3 2C Del36) that can be used for ITC experiments. Dibucaine bound to the wt 2C Del36 protein (Fig. 6C) with an apparent dissociation equilibrium constant (*K_d_*) of 7 μM and a stoichiometric protein/compound ratio (*N*) value of 0.603 ± 0.1 (indicating that one molecule of dibucaine bound to two molecules of protein 2C Del36). Conversely, binding to the 2C Del36 protein carrying mutations that confer partial resistance to dibucaine, 2C(A224V,I227V,A229V), was reduced (*K_d_* of 42 μM), with an *N* value of 0.537 (Fig. 6D). This indicates that dibucaine acts directly by binding to protein 2C Del36 and that the affinity for the protein carrying the resistance mutations is reduced, thus providing a possible explanation for resistance to the compound.

## DISCUSSION

We screened the Prestwick Chemical Library for antivirals against enteroviruses using GFP-expressing CV-B3. Five drugs, fluoxetine, dibucaine, pirlindole, zuclopenthixol, and formoterol, inhibited the replication of CV-B3 >20-fold. We previously showed that fluoxetine inhibits some enteroviruses (i.e., EV-B and EV-D members) by acting on protein 2C (10). The spectrum of antiviral activity and the mode of action of the other four drugs identified in this Prestwick Chemical Library screen were investigated in detail. We found that dibucaine, pirlindole, and zuclopenthixol showed the strongest antiviral effect against EV-B and EV-D members, with dibucaine also showing some activity against EV-A, whereas formoterol inhibited the replication of all EVs and RVs tested. Using Renilla luciferase-expressing CV-B3, time-of-addition assays, and a subgenomic CV-B3 RNA replicon, we showed that all inhibitors studied acted by inhibiting the genome replication stage. Dibucaine and zuclopenthixol were previously identified as inhibitors of CV-B3, but the mechanism of action of these compounds, including the stage in the virus life cycle that was inhibited, had not been explored (9). Here we show that mutations in CV-B3 protein 2C reduce susceptibility to dibucaine, zuclopenthixol, and pirlindole, suggesting that these compounds inhibit replication by targeting 2C. This is further supported by the observation that dibucaine bound to heterologously expressed protein *in vitro*, with a *K_d_* that is in good agreement with the EC_50_ observed for the inhibition of viral replication in cell culture (Fig. 2A and 6C). Binding of dibucaine to the CV-B3 2C mutant protein carrying the resistance mutations was reduced (Fig. 6D), providing a possible explanation for the mechanism by which these mutations confer resistance to the compound. However, the same mutations are responsible for resistance to many compounds belonging to different chemical classes, suggesting that the resistance mechanism might not be a direct binding disruption but that the amino acid changes could just as likely decrease binding by modifying the conformation of the actual binding site. Both scenarios would fit the available experimental data at this point. It should also be kept in mind that not all of the three amino acids Ala224, Ile227, and Ala229 are necessarily involved in binding. Some mutations might have arisen to compensate for the loss of function caused by the mutation(s) required for the loss of sensitivity to the compound. The elucidation of the structure of the protein, preferably in complex with an inhibitor, would significantly add to our understanding of the action of the inhibitor.

The *in vitro* data for dibucaine binding to protein 2C were consistent with a stoichiometric ratio of protein to compound of ∼0.5, equivalent to two molecules of 2C protein and one molecule of dibucaine. In line with data from homologous proteins, the functional form of enteroviral protein 2C is thought to be an oligomer, most likely a hexamer, and there is experimental evidence to support this proposition (25, 26). Differences in the affinities of the compounds for different oligomeric forms could conceivably explain the differences in protein binding observed in this assay. How, then, can the same three mutations confer resistance to these different compounds? This still suggests (to a certain degree) overlapping binding sites, but the conformation of this binding site could adjust upon further oligomerization. Unfortunately, at this point, we cannot test binding to the hexamer in solutions, as no homogenous preparation of 2C hexamer is available.

The apparent *K_d_* for pirlindole in the ITC assay was in the range of 100 μM (data not shown), >10-fold higher than the EC_50_ of replication inhibition in cell culture. Unfortunately, due to the low affinity of pirlindole for protein 2C in this assay, the stoichiometry of the interaction could not be determined.

Previously, MRL-1237, 2-(α-hydroxybenzyl)-benzimidazole (HBB), TBZE-029, hydantoin, and GuHCl were identified as compounds to which mutations in viral protein 2C confer resistance, and now, dibucaine, pirlindole, and zuclopenthixol can be added to this long list. For hydantoin [5-(3,4-dichlorophenyl)methylhydantoin] (27), no data on the sensitivity of the 2C(A224V/I227V/A229V) mutant virus are available, so we do not include this compound in the following discussion. The inhibitors mentioned above belong to a diverse array of chemical classes, with few noticeable similarities for most of them. The compounds also differ in their antiviral spectra. Both TBZE-029 and fluoxetine inhibited EV-B and EV-D but not EV-A, EV-C, or RVs (10, 18; A. de Palma, personal communication). Pirlindole and dibucaine showed strong antiviral activity against EV-B and EV-D, and dibucaine showed some activity against EV-A, but not against EV-C and RVs (Fig. 3). HBB and its derivative MRL-1237 inhibit EV-B and EV-C replication (28, 29), but it is unknown whether they also inhibit EV-A and EV-D (and thereby would act similarly to GuHCl). Interestingly, in spite of their different antiviral spectra, the A224V, I227V, and A229V substitution mutations in protein 2C of CV-B3 confer some resistance to all of these structurally diverse inhibitors (10, 18). Combined with the observation that dibucaine bound directly to protein 2C *in vitro*, we hypothesize that (i) all of these compounds target overlapping binding sites within one virus and (ii) there is a druggable pocket at this position in all of these viruses but (iii) the amino acid composition of this pocket differs between members of different EV species, causing the compounds to bind to some but not all of these pockets. Analysis of the interactions of (some of) these compounds with protein 2C, for example, the elucidation of the protein structure in complex with inhibitors, is highly desirable, as this information could aid the development of broad-spectrum antivirals against this prominent target. While the manuscript was in preparation, Xia et al. (30) showed that, in line with previous predictions based on the presence of conserved sequence motifs (31), 2C of EV-A71 and CV-A16 (both EV-A) possess helicase activity. Unfortunately, pirlindole showed no activity and dibucaine showed only weak activity against EV-A71, and numerous previous attempts to show helicase activity of other enteroviruses failed. The helicase activity would be a plausible target for these compounds. If helicase activity is shown for 2C of an enterovirus, which is sensitive to the described compound, this could be tested.

Compounds that target enterovirus protein 2C were identified in independent screens and are consistently among the top hits (i.e., showing the highest level of inhibition) (9, 10, 32), which enforces the notion that this protein presents a desirable target for antiviral intervention. The frequency with which these compounds are discovered likely reflects a combination of the importance of this protein for the viral life cycle (good drug target) combined with the druggability of this target. Of course, a certain bias due to the limited chemical diversity of available compounds present in the screening libraries cannot be excluded.

In contrast to dibucaine and pirlindole, which acted only on selected enteroviruses, formoterol exhibited activity against a broad spectrum of enteroviruses, including rhinoviruses (Fig. 3). Formoterol, a β2 receptor agonist, was recently reported to decrease titers of RV-B14 replication in human tracheal epithelial cell cultures (11). The authors of that report attributed the decrease in infection to a β2-AR activation-mediated reduction in the expression of intercellular adhesion molecule (ICAM), the cellular receptor of HRV-B14. We observed inhibition of a broad range of enteroviruses; these viruses are known to use not ICAM but another receptor, and importantly, receptor usage also differs for the viruses tested (33). Furthermore, we also observed inhibition of genome replication of the subgenomic replicon (Fig. 4B), which does not rely on entry mediated by these receptors. In summary, downregulation of receptor expression would not explain the antiviral activity of formoterol in our experiments. Another indication that the broad-spectrum antiviral effect is unlikely mediated by β2-AR activation is that salmeterol, another long-acting agonist of β2-AR, had no antienteroviral effect in our experiments (Fig. 5A). Additionally, the inhibition by formoterol could not be counteracted by the addition of the β2-AR antagonist ICI-118,551 (Fig. 5B), an observation that again supports the idea that virus inhibition is not due to β2-AR activation. To identify the target of this new inhibitor, we tested two mutant viruses, the CV-B3 2C(A224V,I227V,A229V) and 3A(H57Y) mutants, which had previously been selected for reduced sensitivity to compounds that inhibit enterovirus genome replication, for resistance to formoterol. Neither of the two mutant viruses was resistant to formoterol (Fig. 6A). Unfortunately, attempts to select for resistant viruses by culturing CV-B3 in the presence of formoterol did not (yet) yield virus with reduced sensitivity to this compound. The failure to recover resistant virus may be due to the fact that we did not observe full inhibition of virus replication by formoterol in a multicycle setting, thus impeding the separation of less sensitive from sensitive viruses (data not shown). To date, we have not been able to elucidate the mechanism of action of this compound. Insight into the mode of action of formoterol could provide a valuable new lead for the development of antivirals, especially since it inhibited a very broad spectrum of activity against enteroviruses, including rhinoviruses. More active analogues could aid in this process if they allowed the selection of resistant mutants, which would provide a starting point for mechanism-of-action studies. However, previous instances where resistance to a compound was not acquired have been described (34, 35). The acquisition of resistance against antivirals by viruses is of course unwanted in a treatment setting. Thus, should enteroviruses prove refractory to the development of resistance against formoterol, this observation would argue for further analysis of this compound.
